# Short-Term and Long-Term Changes of Nasal Soft Tissue after Rapid Maxillary Expansion (RME) with Tooth-Borne and Bone-Borne Devices. A CBCT Retrospective Study.

**DOI:** 10.3390/diagnostics12040875

**Published:** 2022-03-31

**Authors:** Pietro Venezia, Ludovica Nucci, Serena Moschitto, Alessia Malgioglio, Gaetano Isola, Vincenzo Ronsivalle, Valeria Venticinque, Rosalia Leonardi, Manuel O. Lagraverè, Antonino Lo Giudice

**Affiliations:** 1 Department of Medical-Surgical Specialties, Section of Orthodontics, School of Dentistry, University of Catania, Policlinico Universitario “G. Rodolico-San Marco”, Via Santa Sofia 78, 95123 Catania, Italy; pierovenezia@gmail.com (P.V.); serenamoschitto@gmail.com (S.M.); ale.malgioglio@gmail.com (A.M.); gaetano.isola@unict.it (G.I.); vincenzo.ronsivalle@hotmail.it (V.R.); valeria251996@gmail.com (V.V.); rleonard@unict.it (R.L.); 2 Multidisciplinary Department of Medical-Surgical and Dental Specialties, University of Campania “Luigi Vanvitelli”, Via Luigi de Crecchio 6, 80138 Naples, Italy; ludortho@gmail.com; 3 Orthodontic Graduate Program, University of Alberta, Edmonton, AB T6G 2B7, Canada; manuel@ualberta.ca

**Keywords:** rapid maxillary expansion, bone-borne RME, tooth-borne RME, orthodontic, facial aesthetics

## Abstract

The objective of the study was to assess the changes in nasal soft tissues after RME was performed with tooth-borne (TB) and bone-borne (BB) appliances. Methods. This study included 40 subjects with a diagnosis of posterior cross-bite who received tooth-borne RME (TB, average age: 11.75 ± 1.13 years) or bone-borne RME (BB, average age: 12.68 ± 1.31 years). Cone-beam computed tomography (CBCT) was taken before treatment (T0), after a 6-month retention period (T1), and one year after retention (T2). Specific linear measurements of the skeletal components and of the soft-tissue region of the nose were performed. All data were statistically analyzed. Results. Concerning skeletal measurements, the BB group showed a greater skeletal expansion of the anterior and posterior region of the nose compared to the TB group (*p* < 0.05) immediately after RME. Both TB and BB RME induce a small increment (>1 mm) of the alar base and alar width, without significant differences between the two expansion methods (*p* > 0.05). A high correlation was found between skeletal and soft-tissue expansion in the TB group; instead, a weaker correlation was found in the BB group. Conclusion. A similar slight increment of the alar width and alar base width was found in both TB and BB groups. However, the clinical relevance of these differences, in terms of facial appearance, remains questionable.

## 1. Introduction

Rapid maxillary expansion (RME) is the treatment of choice for the correction of transverse maxillary deficiency [1]. RME consists of the separation of the mid-palatal suture, obtained by applying orthopaedic forces through intra-oral devices [2]. The most common design of RME devices is a tooth-borne (TB) expander [3]. Since the TB expander is directly anchored to the teeth, generally the upper first molars, the forces generated by the activation of the appliance can determine undesirable effects on the dentition and alveolar structures [4]. In this regard, common side-effects in TB-RME have been described, such as dental tipping, root resorption, marginal bone loss and reduction in buccal bone thickness [5,6,7], and to moderate these side effects, it has been proposed to support palatal expanders with temporary skeletal anchorage devices (TADs) [8,9]. The skeletal effects and pattern of expansion of TB-RME-RME have been widely documented in the literature [10]; also, recent evidence has suggested that bone-borne (BB) expander could generate greater skeletal expansion compared to TB expander [8].

The effects of RME are not limited to the maxilla but can be extended to the circummaxillary structure as well as several other adjacent structures in the face and the cranium [11,12]. In particular, it can also influence the anatomy and the physiology of the nasal structures [13]. Previous studies [14,15] showed that RME enlarges the dimension of the nasal cavity (about one-third of appliance expansion) and increases its volume by displacing the nasal lateral walls apart. These changes could explain the improvement of nasal breathing and the reduction in nasal airway resistance often recorded in treated subjects [16].

Conversely, the effect of RME on nasal soft tissue has not been deeply investigated, and the few studies available are mostly related to the evaluation of post-treatment changes of surgically assisted RME in adult subjects [17,18]. In this regard, it would be interesting to understand if certain dimensional changes of nasal soft tissue should be expected after RME even in growing subjects, considering that treatment results, including nasal proportions, influence patients’ aesthetic appearance [19]. This aspect is of great clinical relevance considering that transverse skeletal maxillary deficiency is one of the most common skeletal deformities of the craniofacial region among youngsters [20]. In this respect, the aim of the present study was to assess the soft tissue changes of the nose after RME was performed on growing subjects and to evaluate if these changes are different between TB and BB maxillary expanders. For this purpose, we analysed the 3D rendered facial models obtained from cone-beam computed tomography (CBCT) scans of the included subjects. Since BB-RME has shown greater skeletal effects compared to TB-RME [8], we assumed that RME supported by skeletal anchorage (BB-RME) might determine greater soft tissue nasal changes compared to TB-RME, and this assumption was the null hypothesis of the present study.

## 2. Materials and Methods

### 2.1. Study Sample

The research protocol of this retrospective study was approved by the Ethics Review Board of Alberta University (IRB protocol number: Pro00075765) and included a sample of young subjects who completed their orthodontic treatment at the Orthodontic Clinic of the University of Alberta (Edmonton, Canada). Subjects were recruited between September 2019 and August 2021 and randomly assigned to TB-RME or BB-RME. Moreover, the CBCTs used for the present study were obtained from previously published materials [21,22] to avoid unnecessary or additional radiation exposure to the patients. All subjects signed appropriate forms for consent to the treatment.

**Figure 1:**
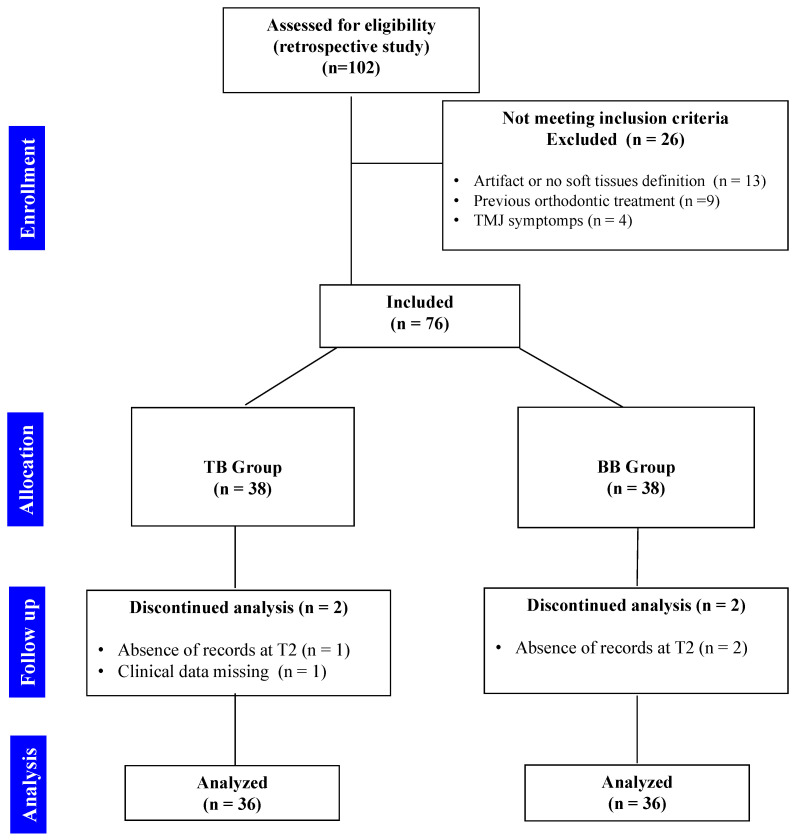
Flowchart showing data recruitment of the present retrospective study.

Inclusion criteria were as follows: (1) age between 11 and 16 years (to avoid extreme differences in the skeletal maturation stage among individuals), registered at the first CBCT acquisition, (2) full permanent dentition (except for the third molar), (3) posterior crossbite, (4) CBCT scans with the field of view (FOV) including all relevant anatomical areas for head orientation and measurements, (5) no artifacts, (6) no temporomandibular joint disorder, (7) no previous orthodontic treatment, (8) no craniofacial anomalies of skeletal and soft-tissue. Figure 1 shows data recruitment process of the present retrospective study.

### 2.2. Treatment

The TB group received a Hyrax appliance designed with bands on the first permanent molars and first premolars. The design of the expander in the BB group includes two mini-screws (length: 12 mm; diameter: 1.5 mm; Straumann GBR System, Andover, MA, USA) inserted in the basal bone at the level corresponding to the area between the permanent first molars and second premolars and joined by a jackscrew.

In both groups, the activation protocol was 0.25 mm/turn with 2 turns per day (0.5 mm/d) in both groups. Expansion screw activations were stopped when overexpansion was achieved, i.e., when the mesiopalatal cusps of the maxillary first permanent molars were in contact with the buccal cusps of the mandibular first permanent molars. The device was maintained for a further 6 months to maintain the results obtained, and no other orthodontic device/therapy was administered to the patient. Parents received a specific form where they reported each activation performed according to the protocol established. The parents of all included subjects had strictly followed the prescription.

### 2.3. Image Acquisition

Cone Beam Computed Tomography (CBCT) was performed before treatment (T0), after 6 months (T1), and one year after retention (T2). Patients were scanned with the same iCAT CBCT unit (Imaging Sciences International, Hartfield, PA, USA). The acquisition protocol was the same for all subjects and included isotropic voxels of 0.3 mm in size, 8.9 s, wide field of view at 120 kV, and 20 mA. The distance between the 2 slices was 0.3 mm.

### 2.4. Skeletal Measurements

**Figure 2:**
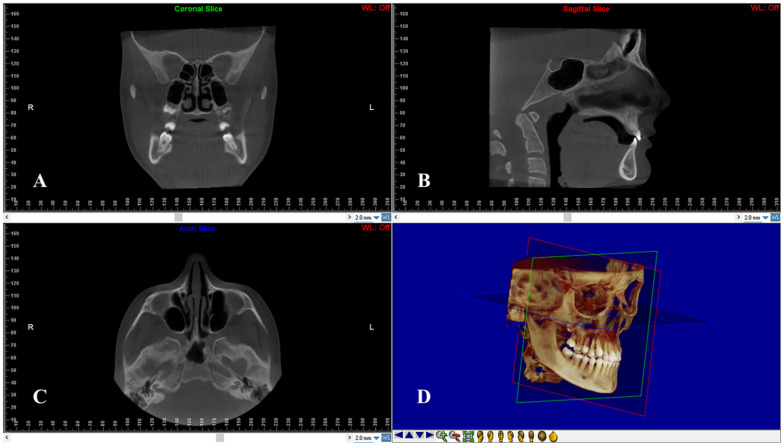
Head re-orientation on coronal (**A**), sagittal (**B**) and axial (**C**) planes of CBCT scans. The 3D image (**D**) shows the head orientation on a 3D space.

On multiple planar reconstruction images, the skull was reoriented to the Frankfort horizontal (FH) as follows (Figure 2): (1) in the frontal view, the mid-sagittal plane was fixed through the center of the anterior nasal spine (ANS), and the axial plane was constructed through both infraorbital skeletal landmarks; (2) in the right sagittal view, the axial plane was placed through the right porion and right infraorbital landmarks. For standardization, the left sagittal view was not processed to avoid orientation problems due to asymmetrically positioned portions; (3) in the axial view, the mid-sagittal plane was constructed through crista Galli and basion [23].

**Figure 3:**
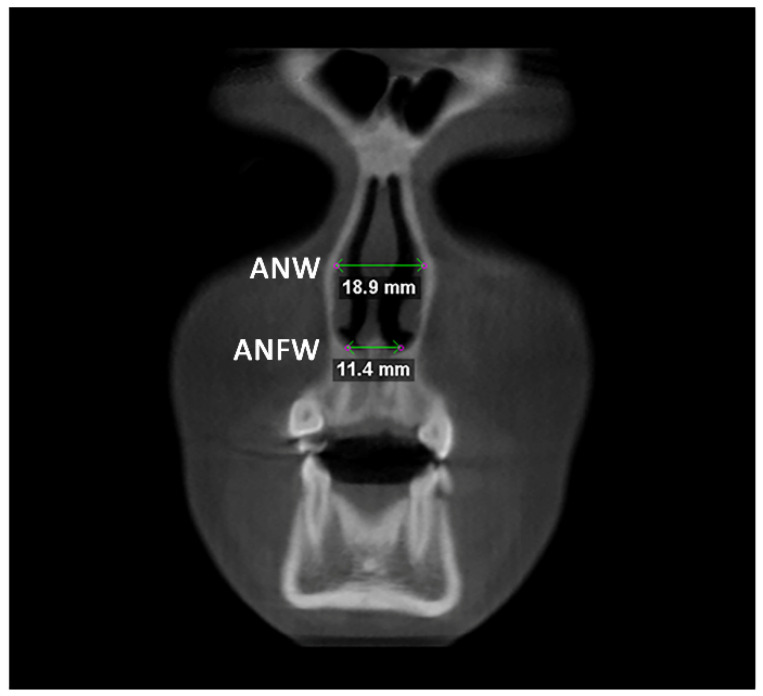
Linear measurements of anterior nasal width (ANW) and anterior nasal floor width (ANFW) in the coronal plane.

**Figure 4:**
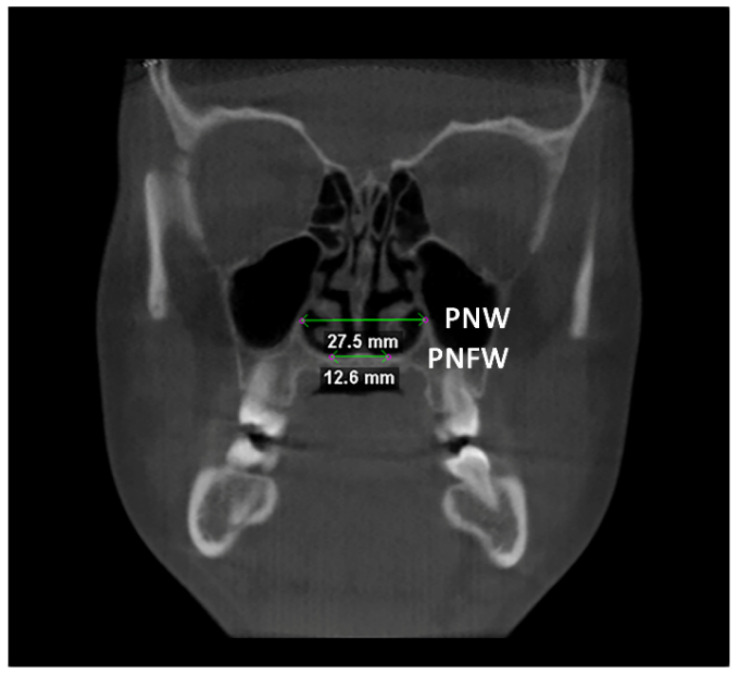
Linear measurements of the posterior nasal width (PNW) and the posterior nasal floor width (PNFW) in the coronal plane.

Afterward, the transverse dimension of the Apertura Piriformis was measured in the anterior and posterior regions. In the coronal plane passing through the cephalometric point N, the linear measurements of anterior nasal width (ANW) and anterior nasal floor width (ANFW) were performed (Figure 3, Table 1). Similarly, in the coronal plane passing through the upper margin of the mesial aspect of the Sella Turcica, the linear measurements of the posterior nasal width (PNW) and posterior nasal floor width (PNFW) were performed (Figure 4, Table 1). The entire procedure for skeletal measurements was performed by using the Dolphin 3D software (Dolphin Imaging, version 11.0, Chatsworth, CA, USA).

### 2.5. Soft Tissue Measurement

**Figure 5:**
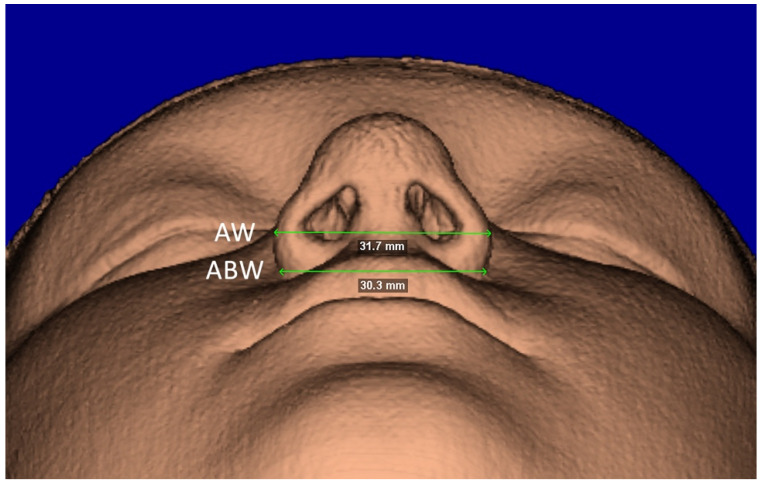
Facial soft-tissue linear measurements of the alar base width (ABW) and the alar width (AW).

**Figure 6:**
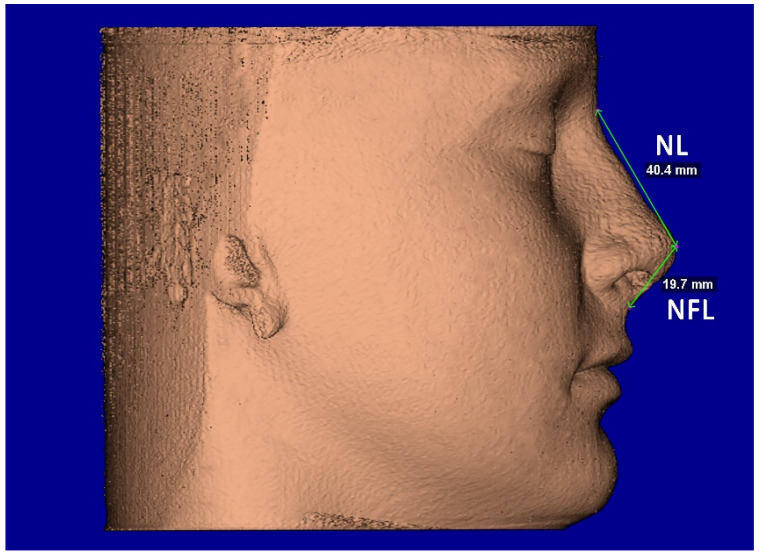
Facial soft-tissue linear measurements of the length of the nose (NL) and length of the nasal filter (NFL).

**Figure 7:**
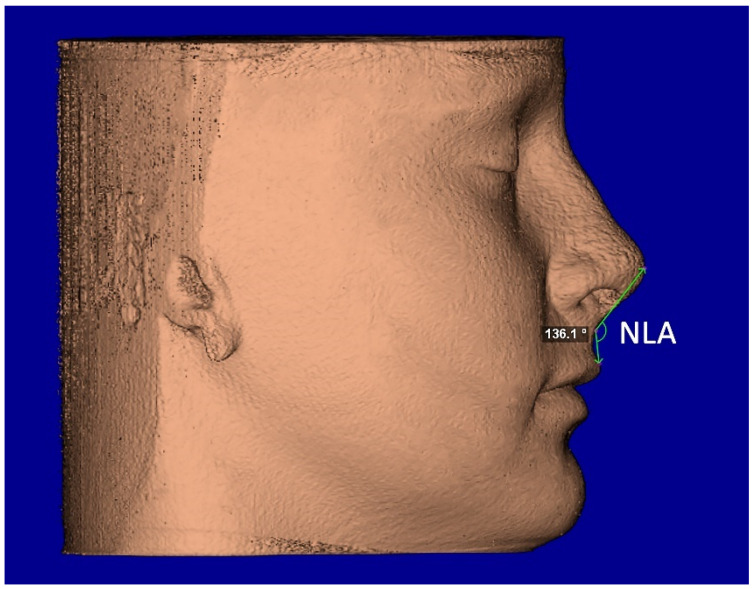
Facial soft-tissue linear measurement of the naso-labial angle (NLA).

The segmentation mask of facial soft-tissue was created, setting the Hounsfield units threshold between −1024 and −200 and then converted into a 3D rendered model. The analysis of the nasal soft-tissue region was performed using the following measurements [17] (Table 1): Alar base width (ABW) (Figure 5), Alar width (AW) (Figure 5), Length of the nose (NL) (Figure 6), Length of the nasal filter (NFL) (Figure 6), Naso-labial angle (NLA) (Figure 7).

The entire procedure for soft tissue measurements was performed by using the Dolphin 3D software (Dolphin Imaging, version 11.0, Chatsworth, CA, USA).

### 2.6. Statistical Analysis

#### 2.6.1. Sample Size Calculation

In the absence of reference data from the literature, calculation of sample size power was preliminary carried out on 20 subjects (10 in the TB group and 10 in the BB group) using the following settings: primary outcome = measurements of ABW parameter, beta error = 0.20, alpha error = 0.05, comparison = difference in the T0-T1 changes of ABW in the TB group, software = SPSS^®^ version 24 Statistics software (IBM Corporation, 1 New Orchard Road, Armonk, New York, NY, USA). The difference detected in the ABW parameter between T0 and T1 was 0.92 mm (SD = 0.88), and the analysis indicated that 28 patients were required to reach 80% power to detect the same difference. However, according to the inclusion criteria, we were able to include 40 subjects which increased the robustness of the data.

#### 2.6.2. Data Analysis

The normal distribution and equality of variance of the data were preliminarily performed with the Shapiro–Wilk Normality Test and Levene’s test. The one-way analysis of variance (ANOVA) and Scheffe’s post-hoc comparisons tests were used for inter-timing assessments; instead, the unpaired Student’s t-test was used for inter-group comparisons. Linear regression analysis was performed to investigate a cause-effect relationship between skeletal and soft-tissue changes, i.e., expansion of the Apertura piriformis (independent variable) and expansion of the alar width and alar base width (dependent variables). A Chi-square test and Student’s *t*-test were used to assess the homogeneous distribution of sex and age variables between the TB and BB groups, respectively.

Ten patients were randomly selected, and the entire procedure was repeated by the same expert investigator (ALG) after 4 weeks. The same patients were also re-measured by a second expert operator (VR). Intra-examiner and inter-examiner reliability for the absolute agreement was assessed for each measurement using the intraclass correlation coefficient (ICC). Data sets were analysed using SPSS^®^ version 24 Statistics software (IBM Corporation, 1 New Orchard Road, Armonk, New York, NY, USA).

## 3. Results

The demographic characteristics of the study sample are reported in Table 2. No differences were found between TB and BB groups concerning sex distribution. However, differences were detected between the two groups according to age distribution; in this regard, subjects in the TB group were about 1 year younger than those included in the BB group.

In both TB and BB groups, there was a statistically significant expansion of the Apertura piriformis (ANW and ANFW) between T0 and T1 (*p* < 0.05), instead no differences were found between T1 and T2 (*p* > 0.05), thus maintaining the post-retention changes (Table 3). The expansion of the Apertura piriformis was significantly greater in the BB group compared to the TB group (TB) (*p* < 0.05) at each time point. The same findings were recorded for the PNW and PNFW measurements (Table 4).

In both TB and BB groups, the alar width (AW) and the alar base width (ABW) slightly increased in both groups between T0 and T1 (*p* < 0.05), while a significant reduction was found at T2, almost reaching pre-treatment values (*p* < 0.05) (Table 3). The increment of the alar width (AW) and the alar base width (ABW) was slightly greater in the BB group compared to the TB group both at 6 months (T0–T1) and 1 year (T0–T2) after maxillary expansion, and such differences were statistically significant (*p* < 0.05) (Table 4).

A small increment of nasal length (NL), nasal filter length (NFL), and nasolabial angle (NLA) were found in both groups between T0 and T1; instead, a small reduction in the same measurements was recorded at T2. However, these changes were not statistically significant (*p* > 0.05) (Table 3). Finally, no differences were found between the TB and BB groups in the changes of NL, NFL, and NLA recorded at 6 months (T0–T1) and 1 year (T0–T2) after maxillary expansion (*p* > 0.05) (Table 4).

A high correlation was found between skeletal and soft-tissue expansion in TB group (from 0.903 to 0.941), instead a weaker correlation was found in the BB group (from 0.695 to 0.742) (Table 5).

Concerning the reliability of the methodology, an excellent correlation was found between intra-operator readings with values ranging from 0.932 to 0.963 for skeletal measurements and from 0.922 to 0.959 for soft-tissue measurements. Inter-operator reliability also showed an excellent correlation between the two readings, with values ranging from 0.901 to 0.916 for skeletal measurements and from 0.915 to 0.928 for soft tissue measurements.

## 4. Discussion

Several studies have demonstrated that RME, both in the form of tooth-borne and bone-borne anchorage systems, increases the transverse dimension and the volume of the nasal cavity, with a consequent potential improvement of the respiratory function [16]. Although the main goal of RME is to correct the skeletal transverse maxillary deficiency and any consequent functional impairment, it would be interesting to understand if this therapy can determine changes in the soft tissue of the nasal region, being that this aspect is relevant from the patients’ aesthetic perspective. To the best of our knowledge, this is the first study in the literature addressing this topic. Previous studies with a similar methodology have been published [17,18]; however, they were focused on changes that occurred after surgically assisted RME, and their findings are far from being comparable to those obtained in the present study, considering the differences between the two treatment approaches. Only two studies have investigated the soft tissue nasal changes after tooth-borne RME using measurements performed on photographic records [24] and in-vivo (clinical inspection using a digital caliper), respectively [25], thus without providing information on the underlying skeletal changes occurring in the tested sample. In this regard, CBCT images allow the analysis of both skeletal and soft tissue changes and perform comparative evaluations, as reported in the present study.

### 4.1. Post-Retention Transverse Changes

Concerning skeletal measurements, the BB group showed a greater skeletal expansion compared to the TB group, which was consistent with previous findings [8]. The TB group showed a greater expansion of the pyriform aperture width compared to the posterior region confirming the wedge-shaped opening of the suture [4]; instead, BB groups showed a more parallel sutural opening [21]. Furthermore, both groups showed a cranio-caudal pattern of expansion (T0/T1 TB: ANW = 1.12 mm, ANFW = 1.61 mm; T0/T1 BB: ANW = 2.01 mm, ANFW = 2.66 mm), confirming the “V” shape opening of the mid-palatal suture [26]. It should be mentioned that subjects in the BB group were slightly older than those included in the TB group (<1 year); thus, they could present an advanced maturational stage of the mid-palatal suture that would have increased the skeletal resistances compared to TB group [27].

Both TB and BB RME induce a small increment (>1 mm) of the alar base and alar width. Such an increment was slightly greater in the TB group with statistical significance; however, it should be considered irrelevant from the clinical perspective. These data are close to those reported by Johnson et al. [25] and were below the increment of 2 mm of the alar base found by Berger et al. [24] with a TB expander. In the latter study, the authors found that the expansion of the soft tissue alar base was in a 1 to 1 ratio with the skeletal increment. Accordingly, in the TB group of the present study, the expansion of the alar base and of the alar width was similar to that of the Apertura piriformis (T0/T1 ANW= 1.12 mm, ANFW = 1.61mm, AW = 1.22 mm, ABW = 1.03 mm), instead, in the BB group, the expansion of the alar base and of the alar width was remarkable below that of the Apertura piriformis (ANW = 2.01 mm, ANFW = 2.66 mm, AW = 1.39 mm, ABW = 1.25 mm). Considering that the transverse skeletal increment was greater in the BB group while both groups showed a similar amount of expansion of the soft tissue, it can be assumed that the response of the soft tissue of the alar region could follow skeletal expansion up to a certain threshold, beyond that further expansion is prevented. Such limitation can be influenced by intrinsic tissue characteristics, such as tension, tone, and thickness of the soft tissue, which may also contribute to the relapse forces. This assumption would be confirmed by the different values of the linear regression between skeletal and soft-tissue expansion found in this study in the TB group (from 0.903 to 0.941) and BB group (from 0.695 to 0.742).

### 4.2. Post-Retention Sagittal Changes

Another assumption of this study is the possibility that RME, in the form of TB and/or BB anchorage, can change the sagittal projection of the soft tissue in the nasal region. A small increment of nasio-labial angle, nasal filter, and nasal length was found in both TB and BB groups; however, these findings were not statistically significant as well as they did not differ between the two groups. As far as we know, the only study that looked at the height of soft tissue in the nose was that of Magnonson et al. [18]. In that study, the authors found an insignificant increase (*p* > 0.05) of 0.18 mm, but in contrast to our study that observed changes after RME, they were observing changes following surgical disjunction. Nevertheless, despite being not statistically significant, the increment of nasio-labial angle, nasal filter, and nasal length data were consistent and could be attributed to adaptive postural changes to accommodate the width and thickness of the expander appliance [24].

### 4.3. Long-Term Changes

One year after appliance removal, all the skeletal and soft-tissue transverse changes obtained after RME were maintained, suggesting that most of the relapse occurred during the retention period, as widely confirmed by literature [10]. Instead, we found a significant reduction in the soft tissue nasolabial angle, nasal length, and nasal filter length, reaching almost pre-treatment values, confirming that the changes recorded during appliance wearing were due to adaptive postural changes of the soft tissue.

Facial aesthetics is a primary concern for patients and clinicians, and consequently, soft-tissue analysis has been integrated into modern orthodontics, being a fundamental aspect of the diagnosis, treatment plan, and decision-making process. Furthermore, in case of documented changes in the facial soft tissue during/after treatment, they should be evaluated and discussed with patients to improve patients’ compliance and confidence in the treatment [28]. In this regard, treatment results including nasal proportions, are considered to have an important influence on patients’ macro-aesthetic appearance [19].

According to the present findings, RME could induce a small increment of the diameter between alar cartilages, and patients with narrow and constrained nasal structures may benefit from the nasal widening effects of surgically assisted rapid maxillary expansion (SARME). Moreover, patients should not be expected to see relevant changes in the nasal soft tissue when undergoing RME assisted by skeletal anchorage. However, the clinical relevance of these findings remains questionable. It is difficult to judge the patients’ perception of the soft tissue changes occurring after RME. There are no established threshold values in the literature for assessing a layperson’s perception of variations in nasal width [29]. Different results may be observed in different patients as a result of the same treatment, with deterioration in one case and improvements in another [18]. In this regard, further studies involving patients’ self-perception of facial changes after RME are recommended to elucidate this aspect; also, studies with long-term follow-up, even using non-invasive 3D imaging digital technology, are warmly encouraged to evaluate soft-tissue behavior years after RME treatment.

### 4.4. Limitations

The study sample consisted of CBCT scans taken with Full Filed of View (FOV), which means that the scans included anatomical areas that are beyond the diagnostic and research interest addressed. In this regard, the usage of ionizing radiations beyond the area of interest should be discouraged according to the ALADAIP principle [30]. However, CBCTs used for the present study were obtained from previously published materials [21,22] to avoid unnecessary or additional radiation exposure to the patients.

The comparative data obtained in the present study may be biased by the different craniofacial skeletal patterns and related muscles characteristics [31], as well as patients’ ages and skeletal maturation stages. Accordingly, caution must be taken in the interpretation of the present findings, and any generalization should be avoided. The absence of matched groups according to the skeletal growth stages, is another limitation of the present investigation. However, growth should not be considered a significant variable in the changes observed in both TB and BB groups, at least between pre-treatment and post-retention stages.

## 5. Conclusions

A similar slight increment of the alar width and alar base width was found in growing subjects treated with TB-RME and BB-RME. However, the clinical relevance of these differences, in terms of facial appearance, remains questionable.

## Figures and Tables

**Table 1:** Description of the linear measurements used in the present study.

	Measurements	Description
**Skeletal Measurements**	**ANW** Anterior Nasal Width	Distance between the most lateral points along the inner surface of nasal lateral walls, taken at the coronal plane passing through point N
	**ANFW** Anterior Nasal Floor Width	Distance between the most lateral points along the inner surface of nasal lateral walls at the nasal floor level, taken at the coronal plane passing through point N
	**PNW** Posterior Nasal Width	Distance between the most lateral points along the inner surface of nasal lateral walls, taken at the coronal plane passing through point S
	**PNFW** Posterior Nasal Floor Width	Distance between the most lateral points along the inner surface of nasal lateral walls at the nasal floor level, taken at the coronal plane passing through point S
**Soft Tissue Measurements**	**AW** Alar Width	Distance between the most lateral points of the alar curvatures on the right (rLAC) and left (lLAC) sides
	**ABW** Alar Base Width	Distance between the right point (rAB) and the left point (lAB) of the facial insertion of the alar base
	**NL** Nasal Lenght	Distance between the soft-tissue N point and PrN points
	**NFL** Nasal Filter Length	Distance between the PrN and SbN points
	**NLA** Nasolabial Angle	Angle between nasal filter and the profile of the upper lip

**Table 2:** Demographic characteristics of the study sample.

Sample Characteristics	Total Sample (n = 40)	TB Group (n = 20)	BB Group (n = 20)	Significance
Sex: male/female	17/23	9/11	8/12	*p* = 0.21 *
Age, y: mean (SD)	12.21 (1.46)	11.75 (1.13)	12.68 (1.31)	*p* = 0.02 **

* *p* value set as ≤0.05. and assessed by chi-square test; ** *p* value set as ≤0.05. and assessed by Student’s *t* test.

**Table 3:** Inferential statistics of measurements calculated before treatment (T0), after 6 months (T1) and one year after treatment (T2).

Measurements	N	Groups	T0		T1		T2		Significance
			Mean	SD	Mean	SD	Mean	SD	
ANW	20	TB	28.01 (b,c)	1.69	29.13 (a)	1.81	29.06 (a)	1.77	*p* = 0.0003
	20	BB	28.32 (b,c)	2.07	30.33 (a)	2.11	30.46 (a)	2.14	*p* = 0.0002
ANFW	20	TB	17.09 (b,c)	2.75	18.7 (a)	2.66	18.5 (a)	2.69	*p* = 0.0003
	20	BB	17.75 (b,c)	1.98	20.41 (a)	1.85	20.53 (a)	1.91	*p* < 0.0001
PNW	20	TB	30.26 (b,c)	2.14	31.1 (a)	1.97	31.02 (a)	2.12	*p* = 0.0072
	20	BB	30.6 (b,c)	4.16	32.56 (a)	3.69	32.25 (a)	4.05	*p* = 0.0001
PNFW	20	TB	25.98 (b,c)	3.46	27.09 (a)	3.77	27.22 (a)	3.57	*p* < 0.0001
	20	BB	26.22 (b,c)	4.10	28.71 (a)	4.23	28.93 (a)	4.37	*p* < 0.0001
AW	20	TB	34.6 (b,c)	2.58	35.82 (a)	2.91	35.22 (a)	3.19	*p* = 0.0035
	20	BB	35.52 (b,c)	3.78	37.11 (a)	4.09	36.57 (a)	3.50	*p* < 0.0001
ABW	20	TB	32.53 (b,c)	3.52	33.56 (a)	3.21	33.6 (a)	3.40	*p* = 0.0004
	20	BB	33.24 (b,c)	3,12	34.49 (a)	3.29	34.66 (a)	3.08	*p* = 0.0002
NL	20	TB	44.45	2.93	44.93	3.27	44.40	3.29	*p* = 0.0881
	20	BB	47.12	5.60	47.65	5.44	47.13	5.28	*p* = 0.0596
NFL	20	TB	18.30	1.83	18.55	1.85	18.32	1.73	*p* = 0.0743
	20	BB	20.17	1.45	20.41	1.55	20.16	1.43	*p* = 0.1315
NLA	20	TB	123.49	8.46	124.10	8.36	123.53	7.60	*p* = 0.0625
	20	BB	130.70	10.09	131.44	10.20	130.80	9.49	*p* = 0.0564

TB = Tooth-Borne group; BB = Bone-Borne group; N = sample number; SD = standard deviation; ANW = Anterior nasal width, ANFW = anterior nasal floor width, PNW = posterior nasal width, PNFW = posterior nasal floor width; AW = alar width, ABW = alar base width, NL = nasal lenght, NFL = nasal filter length, NLA = nasolabial angle. Significance set at *p* < 0.05 and based on one-way analysis of variance (ANOVA) and Scheffe’s post-hoc comparisons tests; a, b, c = identifiers for post-hoc comparisons tests.

**Table 4:** Comparisons of mean changes obtained after 6 months (T0–T1) and one year after treatment (T0–T2) between TB and BB groups.

Measurements	N	Groups	T0–T1			T0–T2		
			Mean	SD	Significance	Mean	SD	Significance
ANW	20	TB	1.12	0.31	*p* < 0.0001	1.05	0.28	*p* < 0.0001
	20	BB	2.01	0.43		2.14	0.37	
ANFW	20	TB	1.61	0.28	*p* < 0.0001	1.41	0.32	*p* < 0.0001
	20	BB	2.66	0.52		2.78	0.53	
PNW	20	TB	0.84	0.21	*p* < 0.0001	0.76	0.25	*p* < 0.0001
	20	BB	1.96	0.27		1.65	0.34	
PNFW	20	TB	1.11	0.19	*p* < 0.0001	1.24	0.24	*p* < 0.0001
	20	BB	2.49	0.51		2.71	0.75	
AW	20	TB	1.22	0.29	*p* = 0.0008	0.62	0.41	*p* < 0.0001
	20	BB	1.59	0.35		1.05	0.31	
ABW	20	TB	1.03	0.17	*p* = 0.0014	1.07	0.15	*p* < 0.0001
	20	BB	1.25	0.23		1.42	0.17	
NL	20	TB	0.48	0.16	*p* = 0.314	−0.05	0.13	*p* = 0.4084
	20	BB	0.53	0.15		0.01	0.17	
NFL	20	TB	0.25	0.21	*p* = 0.872	0.02	0.24	*p* = 0.7823
	20	BB	0.24	0.18		−0.01	0.18	
NLA	20	TB	0.61	0.26	*p* = 0.151	0.04	0.26	*p* = 0.45493
	20	BB	0.74	0.3		0.10	0.36	

TB = Tooth-Borne group; BB = Bone-Borne group; N = sample number; SD = standard deviation. ANW = Anterior nasal width, ANFW = anterior nasal floor width, PNW = posterior nasal width, PNFW = posterior nasal floor width. AW = alar width, ABW = alar base width, NL = nasal length, NFL = nasal filter length, NLA = nasolabial angle. Significance set at *p* < 0.05 and based on Independent Student’s *t* test.

**Table 5:** Linear regression tests model using anterior skeletal changes as independent variables (predictor) and soft tissue changes as dependent variables.

Groups	Predictor Variables	Dependent Variables	R	Coefficients	
				Beta	Standard Error
**TB**	ANW	AW	0.916	0.916	0.020
		ABW	0.903	0.903	0.031
	ANFW	AW	0.927	0.927	0.018
		ABW	0.941	0.941	0.015
**BB**	ANW	AW	0.716	0.716	0.082
		ABW	0.695	0.695	0.102
	ANFW	AW	0,731	0,731	0.079
		ABW	0.742	0.742	0.068

TB = Tooth-Borne group; BB = Bone-Borne group; ANW = Anterior nasal width; ANFW = anterior nasal floor width; AW = alar width; ABW = alar base width.

## Data Availability

Data are available from the corresponding author upon request.
